# Supplementary material for: Weight Categories, Trajectories, Eating Behavior, and Metabolic Consequences during Pregnancy and Postpartum in Women with GDM
Source: Nutrients. 2024 Feb 18;16(4):560. doi: 10.3390/nu16040560 (PMC10892465; doi:10.3390/nu16040560)
Supplement: Supplementary file 1 [file nutrients-16-00560-s001.zip › nutrients-2868197-Supplementary Materials.pdf]

Supplementary Materials

Supplementary Table S1. PPWR at 1 year postpartum according to EGWG.

	EGWG (mean ± SD)	No-EGWG (mean ± SD)	Differences	P-values
**Overall**	6.02±7.40	2.24±5.50	-3.80	<0.0001
**WNW**	6.43±5.57	2.26±4.42	-4.17	<0.0001
**WOW**	6.21±7.20	1.40±6.02	-4.81	<0.0001
**WOB**	4.94±10.02	3.11±7.34	-1.83	0.3087

Data are shown as mean ± standard deviations.

WNW denotes women with normal pre-pregnancy BMI, WOW denotes women with overweight pre-pregnancy BMI, WOB denotes women with obese pre-pregnancy BMI and EGWG denotes excessive gestational weight gain.

P-values are derived from t-tests.

Supplementary Table S2. Weight trajectories in WNW according to BMI tertiles.

	WNW (n=230)						
**Variables**	**T1**	**T2**	**T3**	**Overall P-value**	**P-value T2 vs T1**	**P-value T3 vs T1**	**P-value T2 vs T3**
**GWG**	13.94±5.88	13.57±4.67	13.20±6.21	0.7370	1.000	1.000	1.000
**Weight changes between 6-8 weeks and 1 year postpartum**	-3.55±3.85	-1.62±4.56	-1.67±5.91	0.0184	0.042	0.047	1.000
**PPWR at 1 year postpartum**	2.30±4.09	4.47±5.13	3.08±5.62	0.0249	0.022	0.982	0.262

Data are shown as mean ± standard deviations.
Data are shown for BMI tertiles with the WNW group, T1 being the lowest and T3 the highest tertile.
WNW denotes women with normal weight pre-pregnancy, GWG denotes gestational weight gain and PPWR denotes postpartum weight retention.
P-values are derived from ANOVA.

Supplementary Table S3: Prevalence of glucose intolerance at 1 year postpartum.

Variables	Total (n=464)	WNW (n=230)	WOW (n=138)	WOB (n=96)	P-values
Normal	282 (60.78)	160 (69.57)	75 (54.35)	47 (48.96)	
Prediabetes (n, %)	169 (36.42)	65 (28.26)	59 (42.75)	45 (46.88)	0.004
Diabetes (n, %)	13 (2.80)	5 (2.17)	4 (2.90)	4 (4.17)	

Data are shown as numbers and percentages.
WNW denotes women with normal pre-pregnancy BMI, WOW denotes women with overweight pre-pregnancy BMI, WOB denotes women with obese pre-pregnancy BMI.
P-values are derived from Chi-squared tests for differences in glucose tolerance (normal, prediabetes, diabetes).
